# Epidemiology and Mechanisms of Resistance of Extensively Drug Resistant Gram-Negative Bacteria

**DOI:** 10.3390/antibiotics8020037

**Published:** 2019-04-06

**Authors:** Emily M. Eichenberger, Joshua T. Thaden

**Affiliations:** Department of Medicine, Division of Infectious Diseases, Duke University School of Medicine, Durham, NC 27710, USA; emily.eichenberger@duke.edu

**Keywords:** gram-negative, antibiotic resistance, carbapenem resistant *Enterobacteriaceae*, *Pseudomonas aeruginosa*, *Acinetobacter baumannii*, extensively drug resistant

## Abstract

Antibiotic resistance has increased markedly in gram-negative bacteria over the last two decades, and in many cases has been associated with increased mortality and healthcare costs. The adoption of genotyping and next generation whole genome sequencing of large sets of clinical bacterial isolates has greatly expanded our understanding of how antibiotic resistance develops and transmits among bacteria and between patients. Diverse mechanisms of resistance, including antibiotic degradation, antibiotic target modification, and modulation of permeability through the bacterial membrane have been demonstrated. These fundamental insights into the mechanisms of gram-negative antibiotic resistance have influenced the development of novel antibiotics and treatment practices in highly resistant infections. Here, we review the mechanisms and global epidemiology of antibiotic resistance in some of the most clinically important resistance phenotypes, including carbapenem resistant *Enterobacteriaceae*, extensively drug resistant (XDR) *Pseudomonas aeruginosa*, and XDR *Acinetobacter baumannii*. Understanding the resistance mechanisms and epidemiology of these pathogens is critical for the development of novel antibacterials and for individual treatment decisions, which often involve alternatives to β-lactam antibiotics.

## 1. Introduction

Gram-negative bacteria have a thin peptidoglycan cell wall sandwiched between their inner and outer membranes. This is distinct from the gram-positive bacteria which have a thick peptidoglycan cell wall. Gram-negative bacteria are ubiquitous in nature and cause infection in multiple body sites including the urinary tract, lower respiratory tract, biliary tract, and bloodstream, among others. Due in large part to the selective pressure of antibiotic use, resistance has significantly increased in gram-negative bacteria over the past two decades. This increased resistance has been quite meaningful to patients, clinicians, and the healthcare system generally as antibiotic resistance in gram-negative bacterial infections has been associated with both increased mortality [1,2,3,4] and increased healthcare costs [5,6,7,8] relative to infections with more susceptible bacterial strains.

The adoption of genotyping and whole genome sequencing of large sets of clinical bacterial isolates has greatly expanded our knowledge of how antibiotic resistance emerges. Bacteria have demonstrated a diverse set of mechanisms for degrading antibiotics, modifying the antibiotic target site, or modulating the influx/efflux of antibiotic into or out of the bacterial cell. Understanding the mechanisms and epidemiology of these resistance mechanisms is critical. In the broadest sense, understanding the mechanisms of antibiotic resistance sheds light on how resistance arises and how it is transmitted between bacteria and to patients. The study of resistance mechanisms has also been important in the pharmaceutical industry, as multiple novel agents have emerged to circumvent known resistance mechanisms. These agents have been generally been developed through modification of drug classes that have previously been U.S. Food and Drug Administration (FDA)-approved for antibiotic use. Examples include the novel aminoglycoside plazomicin, novel cephalosporin ceftolozane (which was paired with tazobactam), and novel β-lactamase inhibitors such as avibactam, vaborbactam, and relebactam that were combined with existing β-lactam antibiotics to form drugs such as ceftazidime-avibactam, meropenem-vaborbactam, imipenem-relebactam, and aztreonam-avibactam.

Though routine genotyping to determine bacterial resistance mechanisms is not yet commonplace in the clinical setting, such practice can be useful in certain situations. For example, carbapenem resistant *Enterobacteriaceae* (CRE) containing metallo-β-lactamases (MBL) often have a complex molecular background in which multiple β-lactamases are present [9,10]. Though a single antibiotic may not overcome all resistance mechanisms, knowledge of the particular resistance mechanisms that are present in a bacterial strain can lead to effective directed combination therapy. There is data to suggest that such an approach can be useful in treating MBL-producing CRE [11]. Thus, a thorough understanding of resistance mechanisms in gram-negative bacteria leads to fundamental knowledge of how resistance emerges and transmits, aids in drug discovery, and influences antibiotic therapy in select cases.

In this review we aim to describe the primary mechanisms of resistance and global epidemiology for the most significant antibiotic resistant gram-negative pathogens in the clinical setting: Carbapenem resistant *Enterobacteriaceae*, extensively drug resistant (XDR) *Pseudomonas aeruginosa*, and XDR *Acinetobacter baumannii*. These three pathogens are labeled by the World Health Organization a “critical” threat [12]. Understanding the resistance mechanisms and epidemiology of these pathogens is important, as their treatment often requires therapy with alternatives to β-lactam antibiotics, such as the polymyxins.

## 2. Mechanisms and Epidemiology of Antibiotic Resistance in Carbapenem Resistant *Enterobacteriaceae* (CRE)

### 2.1. Overview of Mechanisms of Resistance in CRE

**Figure 1:**
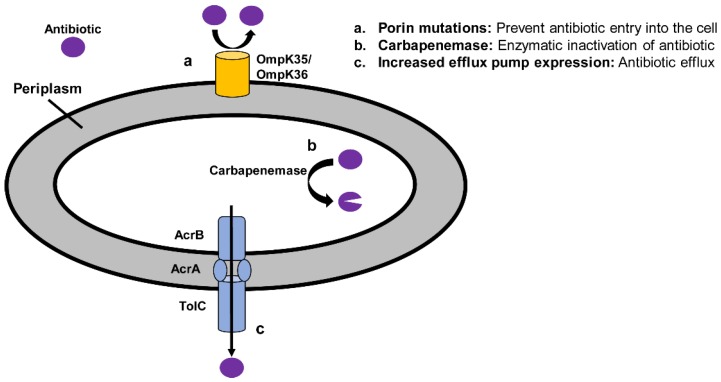
Mechanisms of carbapenem resistance in *Enterobacteriaceae.*

There are three primary mechanisms by which *Enterobacteriaceae* employ resistance to carbapenems: (1) enzymatic degradation through carbapenemase production, (2) expression of efflux pumps, and (3) decreased outer membrane permeability via porin mutations [13]. These mechanisms are discussed in detail in Section 2.3, Section 2.4, Section 2.5, Section 2.6, Section 2.7 and Section 2.8 and illustrated in Figure 1. Carbapenemase expression is a particularly common mechanism of resistance. Carbapenemases and β-lactamases more generally are classified according to their molecular structure with the ambler classification system (Table 1). Carbapenem resistance may emerge through expression of β-lactamases in ambler class A, B, and D, and these enzymes will be discussed more fully in Section 2.3, Section 2.4 and Section 2.5.

### 2.2. Risk Factors for CRE Infection

CRE are a global threat, though, as detailed below, there is great geographic variability in the prevalence and mechanisms of carbapenem resistance. For example, in Europe, CRE infections are endemic in Italy and Greece and sporadic in much of Scandinavia [14]. Significant intranational or regional variability may be present as well. For example, in the U.S., CRE are endemic primarily in the northeast and Great Lakes regions [15]. Therefore, travel to endemic areas is an important risk factor [16]. Additional risk factors generally involve those that increase exposure to antibiotic resistant pathogens such as immune suppression, advanced age, intensive care unit admission, mechanical ventilation, solid organ or hematopoietic transplantation, or prolonged hospital stayes [3,17]. Prior antibiotic use is clearly a risk factor as well [18].

### 2.3. Class A Carbapenemases

Class A carbapenemases (Table 1) require serine at their active site and are able to hydrolyze a broad spectrum of beta-lactams, including penicillins, cephalosporins, and aztreonam [19]. The class A carbapenemases consist primarily of the *Klebsiella pneumoniae* carbapenemase (KPC), *Serratia marcescens* enzyme (SME), and imipenemase/non-metallocarbapenemase-A (IMI/NMC-A). In the U.S., KPC is the most commonly encountered carbapenemase in clinical practice [15]. Several recently FDA-approved antibiotics, including ceftazidime-avibactam, meropenem-vaborbactam, and imipenem-relebactam, contain novel β-lactamase inhibitors that inhibit most KPC enzymes [20,21,22]. The gene coding for KPC, *bla_KPC_*, is located on transferrable plasmids flanked by transposable elements, permitting seamless transference between bacteria of different species [15,23]. In contrast to KPC, the SME and the IMI/NMC-A enzymes are chromosomally encoded [24]. SME has only been found in small sub-population of *Serratia marcescens*, [24] and IMI/NMC-A enzymes have only been identified sporadically in *Enterobacter cloacae* complex isolates [25]. 

The KPC family of enzymes has the most global distribution of all carbapenemases associated with the *Enterobacteriaceae* [15]. The KPC enzyme was first reported in a *K. pneumoniae* clinical isolate in North Carolina, USA, in 1996 [26]. This discovery was soon followed by reports of KPC-producing *Enterobacteriaceae* along the East coast of the United States in the following five years [19,27,28,29,30,31]. As of 2017, KPC containing Enterobacteriacae have been reported in every state in the U.S. [32], and 23 KPC subtypes have been submitted to Genbank (KPC-2–KPC-24; https://externalwebapps.lahey.org/studies/). The incidence of CRE in the U.S. varies highly with geography. For example, in a recent survey from the Centers for Disease Control and Prevention (CDC) involving 7 U.S. institutions, the annual incidence of CRE ranged from 0.35–4.80 annual incident CRE cases/100,000 population [33]. In this study, 47% of the CRE isolates contained a carbapenemase, and all were from the KPC family. Other studies have found the KPC enzyme to be more prevalent, however. For example, in a large study involving 121 cases of CRE bacteremia from 8 medical centers in the New York/New Jersey area, a KPC enzyme was detected in 98 (81% of) cases [34]. KPC-producing *Enterobacteriaceae* are particularly common in the northeastern and upper Midwest regions of the U.S. [15].

KPC was first identified in Europe in 2005 when a patient who had previously been treated at a medical center in New York returned to France [35]. Since that time, KPC-producing *Enterobacteriaceae* have been identified with increasing frequency across Europe and the Mediterranean. KPC-producing *Klebsiella pneumoniae* are now reported to be endemic in Italy and Greece [14]. Studies from these countries have revealed high overall rates of CRE infections that are in large part driven by KPC-producing organisms (80–99% KPC) [36,37].

Emergence of KPC-producing *Enterobacteriaceae* has also been described with increasing frequency in South America, Central America, the Middle East, and Asia. In Latin America, a study of 255 patients with *Enterobacteriaceae* bacteremia in 11 hospitals from 7 countries (Argentina, Colombia, Ecuador, Guatemala, Mexico, Peru, Venezuela) revealed that 21% of cases were from carbapenemase-producing bacteria. The majority of these (83%) were KPC-producing *Enterobacteriaceae*. In Israel, a national intervention to curb CRE infections, driven primarily by KPC-producing bacteria, led to a decrease in CRE acquisitions/100,000 patient days from 55.5 in 2008–4.8 in 2012 [38]. In Asia, KPC-producing CRE are particularly prevalent in China. A study of 109 carbapenem resistant *K. pneumoniae* bacteremia isolates at a China teaching hospital revealed that 71% were KPC-producers [39]. There is little data on CRE epidemiology in Africa.

Molecular epidemiology studies indicate that the global spread of KPC-producing *Enterobacteriaceae* is in large part due to the clonal expansion of *K. pneumoniae* sequence type (ST) 258 [40,41,42]. ST258 has been shown to consist of two genetic clades with distinct capsule polysaccharide gene regions [43,44]. Interestingly, ST258 clade I is more associated with subtype KPC-2, while ST258 is more associated with subtype KPC-3 [45]. Given the clinical importance of ST258, multiple groups are developing immunotherapeutics directed against *K. pneumoniae* ST258 [46,47]. However, there is significant regional variability in the molecular epidemiology of KPC-producing *K. pneumoniae,* as a survey of 111 KPC-producing *K. pneumoniae* isolates from throughout Spain in 2012–2014 did not identify a single ST258 strain [48]. Interestingly, this study found that single-locus variants of ST258, including ST11 (associated with KPC-2) and ST512 (associated with KPC-3), as well as the unrelated ST101 (associated with KPC-2), were the most prevalent KPC-producing *K. pneumoniae*.

The first SME-producing *Serratia marcescens* was found in England from a clinical isolate collected in 1982 [49] and has since been infrequently identified across the U.S. and the United Kingdom [50]. The IMI/NMC-A enzymes appear to be related to SME-1, with 70% amino acid identity [51], and have been detected in *Enterobacter cloacae* complex isolates in France, Argentina, and the U.S. [52].

### 2.4. Class B Carbapenemases

The Ambler Class B carbapenemases, referred to as metallo-β-lactamases (MBLs), utilize zinc as an essential cofactor in cleaving the β-lactam ring [53]. The class B MBLs hydrolyze all β-lactams, save for the monobactams (i.e., aztreonam) [11,54]. MBLs are not hydrolyzed by β-lactamase inhibitors that are commonly encountered in the clinical setting such as clavulanate, tazobactam, or avibactam, and instead are inhibited by metal chelating agents such as ethylenediaminetetraacetic acid (EDTA) which are not available for clinical use [53]. However, pipeline agents, including the novel cephalosporin cefiderocol and the monobactam-β-lactamase inhibitor combination aztreonam-avibactam, have demonstrated good in vitro efficacy against MBL-containing CRE [55,56]. The class B MBLs can be further categorized based on whether they are encoded by transferrable elements versus chromosomes. The notable transmissible MBLs in *Enterobacteriaceae* are the Verona integron-encoded metallo-β-lactamase (VIM), IMP (for ‘active on imipenem’), and New Delhi metallo-β-lactamase (NDM) enzymes. 

A substantial burden of class B carbapenemase-producing *Enterobacteriaceae* is in Asia. For example, the carbapenemase NDM is endemic in India, Pakistan, and Bangladesh [57]. It is named for New Delhi where it was first isolated from a Swedish patient infected with *K. pneumoniae* who had previously received health care in New Delhi, India, in 2008 [58]. Studies from India and Pakistan have demonstrated that 8–12% of all *Enterobacteriaceae* harbor an NDM enzyme, with little detection of non-NDM carbapenemases [59,60,61]. Worryingly, studies from these countries have also revealed that NDM-containing *Enterobacteriaceae* often contained additional β-lactamases (such as the CTX-M [so named for its ‘activity against cefotaxime’ and isolation from Munich]) that confers the extended-spectrum β-lactamase (ESBL) phenotype) [62] and are commonly isolated from environmental samples such as tap water and sewage effluent [63]. Furthermore, these environmental samples revealed spread of *bla*_NDM-1_ (i.e., the gene for NDM-1) to novel bacterial species including *Shigella boydii* and *Vibrio cholerae* [63]. NDM-producing *Enterobacteriaceae* have also been identified with increasing frequency in the UK as well as across Europe, with notable interregional spread in Denmark, Romania, and Poland [14]. NDM-producing bacteria are less common in Canada, South America, and the U.S. [15]. As of 2017, 35 US states have reported NDM-producing *Enterobacteriaceae* with a cumulative incidence of 379 cases [32]. The VIM carbapenemase is the most frequently reported MBL worldwide, though is relatively rare in *Enterobacteriaceae* [54,64]. VIM-producing *Enterobacteriaceae* are largely found in Italy, Greece, Spain, and Hungary [65].

### 2.5. Class D Carbapenemases

The class D serine-carbapenemases consist of the OXA β-lactamases, some of which are able to hydrolyze carbapenems. The most commonly encountered class D β-lactamase in *Enterobacteriaceae* are the OXA-48-like enzymes, which weakly hydrolyze carbapenems and typically spare the expanded spectrum cephalosporins [66]. OXA-48-like producing *Enterobacteriaceae* were first identified in Turkey in 2001 [67] and have reached endemic levels in Turkey and in Malta based on assessments by national experts [14]. Recent studies from Turkey showed that 69–92% of carbapenemases were OXA-48 [68,69]. OXA-48-like-producing *Enterobacteriaceae* have spread to neighboring countries in Europe, with high levels of interregional spread in Belgium, France, Romania, and Spain [14]. OXA-48-like producing *Enterobacteriaceae* are uncommon in the U.S. [32].

### 2.6. Efflux Pumps

While enzymatic degradation of antibiotic by carbapenemases is the primary mechanism of carbapenem non-susceptibility in *Enterobacteriaceae*, additional mechanisms, such as efflux pumps and porin mutations, may also play a role (Figure 1). Efflux pumps belonging to the resistance-nodulation-division (RND) family are clinically significant mechanisms of resistance in gram-negative bacteria. One such pump is the AcrAB-ToIC pump found in *Enterobacteriaceae*. It is tripartite complex that spans the inner membrane, the periplasm, and the outer membrane in order to expel antibiotics from the cell. The AcrAB-TolC pump may confer resistance to other antibiotics as well, such as other β-lactam antibiotics, macrolides, tetracycline, and fluoroquinolones, among others [70]. The extent to which the upregulation of efflux pumps influences the global epidemiology of carbapenem resistant Enterobacteriaceae (CRE) is not clear. 

### 2.7. Porin Mutations

Mutations in porins such as OmpK35 and OmpK36 alone do not generally result in carbapenem resistance, though can achieve such when present in AmpC or CTX-M producing *Enterobacteriaceae* [71,72,73,74] (Figure 1). CTX-M and AmpC enzymes possess low levels of carbapenem hydrolytic activity. In some cases, the combination of high β-lactamase expression and decreased porin expression creates an “antibiotic trapping phenomenon” whereby the carbapenem is “trapped” by the β-lactamase through irreversible binding, but not degraded [71]. In addition, the presence of *ompK36* porin gene mutations in KPC-producing *K. pneumoniae* strains has been associated with high-level carbapenem resistance and attenuated responses to carbapenem–colistin therapy [75,76,77]. The contribution of porin mutations in the global epidemiology of CRE is not well understood, though in the U.S. a multicenter study revealed *ompK35* and *ompK36* porin mutations in 84% and 34% of carbapenem-resistant *K. pneumoniae,* respectively [34].

### 2.8. Colistin Resistance

Unfortunately, *Enterobacteriaceae* strains which are resistant to carbapenems frequently acquire resistance to other classes of antibiotics, including aminoglycosides, tetracyclines, trimethoprim-sulfonamides, and fluoroquinolones [78]. Infections with such highly resistant *Enterobacteriaceae* commonly require therapy with a polymyxin, though even this is potentially complicated by the emergence of polymyxin resistance. Colistin is a polycationic peptide that displaces cations in the lipopolysaccharide (LPS) component of bacterial outer membrane, leading to disruption of the outer membrane and cell death [79]. Colistin resistance can emerge through multiple mechanisms including increased capsule production (which decreases binding of colistin to its target LPS), loss of LPS, and modification of LPS [80]. Unfortunately, colistin resistance can be transmitted between bacteria by way of the plasmid-encoded *mcr-1,* which modifies the antibiotic target site and decreases polymyxin binding [81].

## 3. Mechanisms and Epidemiology of Antibiotic Resistance in XDR *P. aeruginosa*

### 3.1. Overview of Mechanisms of Resistance in XDR P. aeruginosa

**Figure 2:**
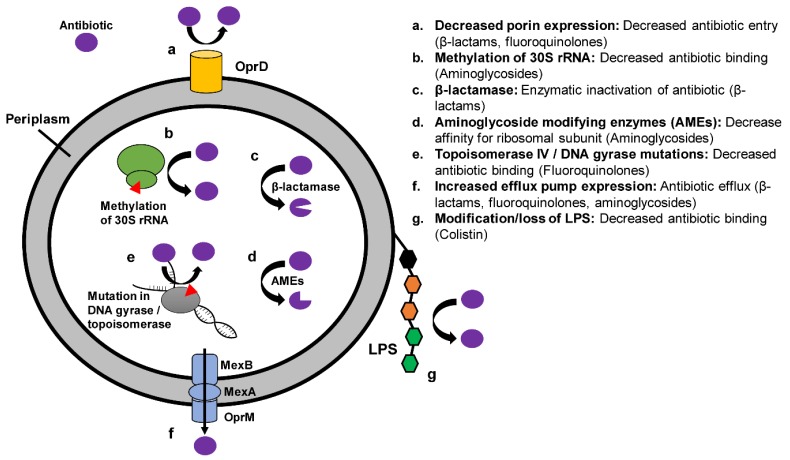
Mechanisms of antibiotic resistance in *P. aeruginosa.*

*P. aeruginosa* is intrinsically resistant to multiple antibiotics including rifampin, tetracycline, chloramphenicol, trimethoprim-sulfamethoxazole, and many β-lactams [82]. Low membrane permeability and efflux pump expression are partly responsible for this intrinsic resistance [82]. Additional resistance mechanisms may be present and drive highly antibiotic resistant phenotypes such as XDR *P. aeruginosa*, which is defined as susceptible to only one or two classes of antipseudomonal antimicrobials [83,84]. There are four general mechanisms by which *P. aeruginosa* generate resistance to antipseudomonal antimicrobials: (1) efflux pumps, (2) porin expression, (3) antibiotic target mutations, and (4) drug-inactivating enzymes [85] (Figure 2). It is the combination of these mechanisms which results in the XDR phenotype. Mechanisms of antibiotic resistance in *P. aeruginosa* are discussed in detail in Section 3.3, Section 3.4, Section 3.5 and Section 3.6.

### 3.2. Prevalence of and Risk Factors for XDR P. aeruginosa

Study of the epidemiology of XDR *P. aeruginosa* is complicated by a lack of global surveillance data and the diverse mechanisms by which *P. aeruginosa* develops resistance. The global emergence of XDR *P. aeruginosa* is due to the accumulation of multiple unrelated resistance mechanisms [85]. In the U.S., the international network for optimal resistance monitoring (INFORM) group evaluated *P. aeruginosa* isolates across 79 U.S. medical centers and found that 9% of all isolates exhibited the XDR phenotype. A similar or higher prevalence of the XDR phenotype among clinical *P. aeruginosa* clinical isolates has been found in epidemiological studies from Spain (11%) [86], Thailand (22%) [84], Greece (25%) [87], and Iran (33%) [88]. In some patient populations, the XDR phenotype may occur at an alarmingly high rate, as in *P. aeruginosa* from burn patients in a single center study in Iran (87%) [89] or solid organ transplant patients from a single center study in Spain (63%) [90]. Risk factors for acquisition of XDR *P. aeruginosa* infections, relative to more susceptible *P. aeruginosa* infections, include receipt of total parenteral nutrition [84], prior fluoroquinolone use [84,87], prior carbapenem use [84], hematological malignancy [87], mechanical ventilation [91], and Acute Physiology and Chronic Health Evaluation II (APACHE-II) score [91]. 

### 3.3. Efflux Pumps

Efflux pumps contribute to antibiotic resistance of multiple drug classes (Figure 2). For example, mutations leading to upregulation of efflux pumps such as the MexA-MexB-OprM system can lead to increased resistance to anti-pseudomonal β-lactams, fluoroquinolones, and aminoglycosides [92,93,94]. When upregulation of efflux pump expression is present in combination with targeted outer membrane mutations, additional resistance may be achieved [95]. In vitro overexpression of *mexAB-oprM* is readily achieved under selection pressure from antibiotic exposure, and often stems from mutations in the transcription factors that regulate *mexAB-oprM* expression [96,97]. The global epidemiology of *mexAB-oprM* expression and its influence on the *P. aeruginosa* XDR phenotype is not well understood.

### 3.4. Porin Mutations

OprD is a porin protein which allows passive uptake of basic amino acids across the outer membrane, as well as uptake of carbapenems [93]. Loss of OprD confers resistance to imipenem and reduced susceptibility to meropenem [93] (Figure 2). When the loss of OprD is combined with upregulation of MexA-MexB-OprM, resistance to meropenem, imipenem, ceftazidime, ureidopenicillins, carboxypenicillins, quinolones, tetracycline, and chloramphenicol is achieved [93]. OprD mutation is particularly associated with the “high risk” *P. aeruginosa* clone ST75 (see Section 3.6 below)

### 3.5. Modification of Antibiotic Target Site

While fluoroquinolone resistance is commonly driven by changes in efflux pump expression (Section 3.3), mutations in the fluoroquinolone targets *parC* and *gyrA* have been identified in *P. aeruginosa* strains which confer resistance to fluoroquinolones [98]. These genes code for topoisomerase IV and DNA gyrase, respectively, and are critical in bacterial DNA replication [99,100]. Aminoglycosides exert their antibacterial effect through binding and inhibiting the bacterial 16S rRNA, and resistance to aminoglycosides in *P. aeruginosa* may emerge through modification of the antibiotic target 16S rRNA. For example, 16S rRNA methylases RmtA, RmtD, and ArmA have been described in *P. aeruginosa* and may confer resistance to all clinically useful aminoglycosides [101,102,103]. Colistin resistance in *P. aeruginosa* may emerge through mutation of multiple genes such as *pmrAB* and *phoPQ*, among others, with the final common pathway being the modification of the LPS target of colistin [104].

### 3.6. Antibiotic Degradation

Upregulation or acquisition via horizontal transfer of drug-inactivating enzymes contribute to antimicrobial resistance in *Pseudomonas. P. aeruginosa* intrinsically possess class C AmpC enzymes, which are chromosomally encoded cephalosporinases (Table 1). Mutations causing increased expression of AmpC enzymes, or mutations which enable hyper-inducible AmpC production, result in resistance to penicillins, monobactams, and cephems [73]. Additionally, horizontal acquisition of a wide variety of β-lactamases from classes A, B, and D may result in a wide spectrum of resistance. Some of these β-lactamases are associated with particular “high risk” *P. aeruginosa* clones that have a wide global distribution (Section 3.7 below). Acquisition of narrow spectrum β-lactamases such as PSE (*Pseudomonas* specific enzyme)-1, PSE-4, and some OXA-type enzymes enable resistance to the antipseudomonal penicillins and cefoperazone but do not have activity against monobactams, carbapenems, or antipseudomonal cephems (Table 1) [82]. Acquisition of broad-spectrum beta-lactamases such as PER (*Pseuomonas aeruginosa* RNL-1)-1, VEB (Vietnamese extended-spectrum β-lactamase)-1, GES (Guiana extended spectrum)-1, GES-2, and some OXA-type enzymes confer resistance to anti-pseudomonal penicillins, anti-pseudomonal cephems, and monobactams, but not carbapenems [82]. Finally, acquisition of MBLs including IMP, SPM (Sao Paulo metallo-β-lactamase), GIM (Germany imipenemase), and VIM enzymes can confer resistance to all antipseudomonal β-lactams, save for the monobactams [52,82,105]. 

Aminoglycoside modifying enzymes can similarly be acquired via horizontal gene transfer. The aminoglycoside modifying enzymes include enzymes which may phosphorylate, adenylate, and acetylate the aminoglycoside, thereby decreasing its affinity to bind the ribosomal subunit [85]. The most frequent aminoglycoside modifying enzyme in *P. aeruginosa* is AAC(6’)-II which mediates resistance to tobramycin, gentamicin, and netilmicin, and ANT(2”)-I, which mediates resistance to gentamicin and tobramycin [106]. The novel aminoglycoside plazomicin retains activity against these aminoglycoside-modifying enzymes through structural modification of the antibiotic [107].

### 3.7. Clonal Structure of XDR P. aeruginosa

*P. aeruginosa* demonstrates significant genomic variability, though isolates with the XDR phenotype display a more clonal structure [86,108,109]. For example, in one study, 90% of all XDR *P. aeruginosa* isolates belonged to one of three “high risk” clones: ST111, ST175, or ST235 [109]. Of these, ST235 has the most global distribution and has been detected on five continents [110]. ST111 is also globally distributed, with the exception of Oceania, while ST175 is uncommon outside Europe [110].

Even among *P. aeruginosa* clinical isolates within a single high-risk clone, the resistance mechanisms driving the XDR phenotype are remarkably diverse. In ST235, for example, 39 different β-lactamases from classes A, B, and D have been identified [110]. Class A enzymes such as the ESBLs PER-1 and GES enzymes are particularly widespread [111,112,113]. Class B carbapenemases are more common among ST235 isolates, and VIM-1 is the most geographically widespread [85,111]. Of concern is the discovery of ST235 *P. aeruginosa* isolates containing the KPC-2 enzyme in Colombia [111,114].

*P. aeruginosa* ST111 also contains an array of β-lactamases, albeit fewer than those belonging to ST235 [110]. The most widespread β-lactamase in ST111 isolates is the class B enzyme VIM-2 [86,111,114,115], though class A enzymes including the ESBLs, GES, and VEB enzymes may be detected as well [111,116]. As with ST235, *Klebsiella pneumoniae* carbapenemase-2 (KPC-2) containing ST111 have been detected in Colombia [115].

*P. aeruginosa* ST175, in contrast to ST235 and ST111, often generates the XDR phenotype through OprD porin activation, AmpC hyperproduction, and efflux pump overexpression [86]. As with ST235 and ST111, however, the VIM-2 enzyme is commonly present [117,118].

## 4. Mechanisms and Epidemiology of Antibiotic Resistance in XDR *A. baumannii*

### 4.1. Mechanisms of Resistance in XDR A. baumannii

**Figure 3:**
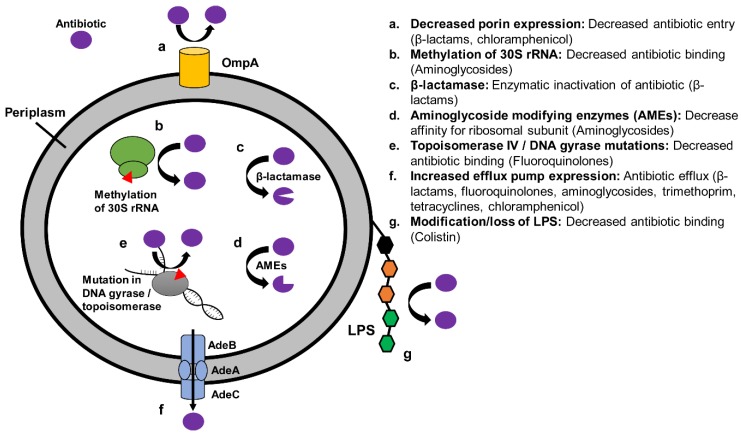
Mechanisms of antibiotic resistance in *A. baumannii.*

*A. baumannii* is a nosocomial and opportunistic pathogen that is often subjected to significant selective pressures from antibiotics in the hospital environment. This may result in chromosomal mutations and acquisition of resistance genes via horizontal transfer to ensure its survival and spread. *A. baumannii* is intrinsically resistant to several groups of antimicrobials, including glycopeptides, lincosamides, macrolides, and streptogamins. XDR *A. baumannii* may arise through a variety of mechanisms, including efflux pumps, porin expression, antibiotic target mutations, and drug-inactivating enzymes (Section 4.3, Section 4.4 and Section 4.5) (Figure 3).

### 4.2. Prevalence of and Risk Factors for XDR A. baumannii Infection

*A. baumannii* is an important cause of healthcare-associated infections. Determining the prevalence of XDR *A. baumannii* is challenging, in that large multicenter studies investigating antibiotic resistance in *A. baumannii* may not address the XDR phenotype per se. However, one of the hallmarks of XDR *A. baumannii* is carbapenem resistance, as carbapenem resistant isolates are often XDR [119]. According to the National Healthcare Safety Network at the centers for disease control in the US, the overall rate of carbapenem resistant *A. baumannii* was 47% in central line-associated bacteremia and 64% in catheter-associated urinary tract infections [120]. In Europe, a recent report from the European center for disease prevention and control showed that 49% of *A. baumannii* (2861/5853) were carbapenem resistant [121]. There was significant international variability, with countries in southern Europe and the Baltic exhibiting particularly high resistance [121]. The prevalence of carbapenem resistant *A. baumannii* is similarly high in other parts of the world including southern and southeast Asia (40–60%) [122], and Latin America (40–80%) [123]. Risk factors for colonization and/or infection with XDR *A. baumannii* include prior antibiotic use (specifically carbapenem, third generation cephalosporin, or fluoroquinolone use), indwelling central line, mechanical ventilation, tracheostomy, recent surgery, and intensive care unit (ICU) stay [124,125]. 

### 4.3. Outbreaks of XDR A. baumannii Infection

Outbreaks of XDR *A. baumannii* often occur in an ICU setting. The selective pressure of broad-spectrum antibiosis in ICUs and the ease and rapidity of acquiring multiple antibiotic resistance mechanisms are drivers for high antibiotic resistance. The ability of *A. baumannii* to survive on inanimate surfaces for extended periods of time plays a role as well. For example, *A. baumannii* was isolated from hospital bed rails nine days after the infected patient was discharged from the hospital [126]. Multiple ICU outbreaks have been traced back to a contamination source such as respiratory equipment or the hands of healthcare workers [127]. *A. baumannii* ICU outbreaks have been reported in cities across Europe, South America, Africa, Asia, the Middle East, and the U.S. [125,128,129,130,131,132,133,134,135]. Additionally, interinstitutional and international spread of such outbreaks have been described in cases of colonized or infected patients who are transferred to another healthcare facility [125,136,137]. Community acquired XDR *A. baumannii* infections are exceedingly rare [138,139].

### 4.4. Efflux Pumps and Decreased Outer Membrane Permeability

Increased efflux pump activity is a key mechanism of resistance in *A. baumannii*. Specifically, overexpression of the *adeABC* resistance-nodulation-cell division (RND) efflux system enables resistance to several classes of antibiotics, including fluoroquinolones, aminoglycosides, trimethoprim, tetracyclines, and chloramphenicol [140] (Figure 3). When the *adeABC* efflux pump is over-expressed in *A. baumannii* harboring carbapenem-hydrolyzing oxacillinases, high level carbapenem resistance is achieved [141]. An additional efflux system-*adeIJK* may confer resistance to tigecycline. This is particularly the case when *adeIJK* and *adeABC* are co-expressed [142]. Efflux pumps encoded by the *tet* genes are typically associated with mobile genetic elements. TetA confers resistance to tetracycline, while TetB confers resistance to minocycline [143]. The outer membrane protein OmpA is a major *A. baumannii* porin. Mutations targeting the *ompA* gene may result in decreased outer membrane permeability, and achieve resistance to chloramphenicol, aztreonam, and nalidixic acid [144] (Figure 3).

### 4.5. Mutations in Antibiotic Target

Resistance to fluoroquinolones may emerge through mutations in *gyrA* and *parC,* subunits of DNA gyrase and DNA topoisomerase, respectively, which decrease the affinity of fluoroquinolones for their targets [145,146] (Figure 3). An international collection of fluoroquinolone-resistant *A. baumanii* has demonstrated that mutations in genes *gyrA* and *parC* are highly prevalent [147]. The role that additional mechanism of resistance, such as overexpression of efflux pumps, plays in the global epidemiology of fluoroquinolone resistance is not known. Mutations in enzymes involved in the biosynthesis of lipopolysaccharide (LPS) leading to the modification or loss of the lipid A portion of LPS may lead to colistin resistance through decreased colistin binding [148,149].

Aminoglycoside-modifying enzymes including acetyltransferases, nucleotidyltransferases, and phosphotransferases may be acquired via plasmid exchange, transposons, or class 1 integrons [150,151] (Figure 3). These enzymes may occur in isolation or in combination to enable resistance to aminoglycosides [150]. As described in Section 3.5, aminoglycosides bind and inhibit the bacterial 16S rRNA. Modification of the target 16S rRNA is another effective mechanism of resistance (Figure 3). For example, 16S rRNA methyltransferases have been described in *A. baumannii* and are responsible for high level resistance to all aminoglycosides, including amikacin [152]. ArmA is one such 16S rRNA methyltransferase that is well characterized in *A. baumannii* and confers resistance via interfering with the binding of aminoglycosides to their site of action [152]. ArmA has been detected worldwide [152,153,154,155,156]. The global epidemiology of the additional 16S methylases (e.g. RmtA, RmtB, RmtC, RmtD, RmtE, RmtF, RmtG, NpmA) is less clear. Of note, isolates harboring 16S rRNA methylases typically also harbor ESBLs or MBLs by nature of their location on transferable plasmids and association of transposable structures [157]. 

### 4.6. Antibiotic Degradation

*A. baumannii* intrinsically possess a chromosomal AmpC cephalosporinase, which hydrolyzes cephalosporins at a low level. Similarly, *A. baumannii* intrinsically possess a class D oxacillinase, OXA-51-like enzyme, which hydrolyze penicillins and carbapenems at a low level. Insertion of a strong transcriptional promotor, *ISAba1*, upstream of the *ampC* cephalosporinase or OXA-51-like gene results in clinically significant cephalosporin resistance [158,159]. In addition, acquisition of carbapenemases have been well described in *A. baumannii*. Specifically, class D enzymes such as OXA-23-, OXA-40-, and OXA-58-like enzymes are the most commonly detected carbapenemases in *A. baumannii* and may be plasmid or chromosomally encoded [157]. OXA-23-like enzymes are the most widespread carbapenem-hydrolyzing enzymes detected in *A. baumannii* worldwide and are common drivers of nosocomial outbreaks of carbapenem-resistant *A. baumannii* [157,160]. These enzymes are typically associated with transposons Tn2006 and Tn2007 [161,162,163]. Other class D β-lactamases with carbapenemase activity have more regional distributions such as OXA-25, OXA-26, and OXA-40, which are predominantly detected in Europe [164,165] and OXA-72 which is predominantly detected in Asia [166,167,168,169]. Less common causes of carbapenem resistance in *A. baumannii* include the class A and B β-lactamases. Class A KPC-producing *A. baumannii* are exceedingly rare and have mostly been described in a series of isolates from Puerto Rico [170]. Class B IMP-, VIM-, SIM- (Seoul imipenemase), and NDM-type enzymes have been detected in *A. baumannii,* though the IMP- and VIM-type enzymes are most commonly isolated and widely distributed [171]. 

## 5. Conclusions

Genotyping and whole genome sequencing of highly resistant gram-negative bacteria has revealed many of the diverse ways that these bacteria develop antibiotic resistance. This fundamental knowledge has impacted our understanding of how resistance emerges, how it is transferred to other bacteria and to patients, the development of novel antibiotics, and treatment decisions in select patients. Further integration of bacterial genotyping for resistance mechanisms into the clinical setting will likely impact our therapeutic decisions in meaningful ways.

## Figures and Tables

**Table 1:** Ambler classification of β–lactamases.

Ambler Class	β-Lactamases	Active Site Agent	Examples	Substrates
A	Penicillinases	Serine	PSE TEM, SHV, CTX-M, VEB, PER, GES KPC, SME, IMI/NMC-A	Penicillins Penicillins, 3rd generation cephalosporins All β-lactams
B	Metallo-β-lactamases	Zinc	IMP, VIM, NDM, SPM, GIM	All β-lactams, except monobactams
C	Cephalosporinases	Serine	AmpC	Cephamycins, 3rd generation cephalosporins
D	Oxacillinases	Serine	OXA	All β-lactams, though class D enzymes have highly variable spectra of activity

Abbreviations: CTX-M, active against cefotaxime (CTX) and isolated in Munich (-M); GES, Guiana extended spectrum; GIM, German imipenemase; IMP, active on imipenem; KPC, Klebsiella pneumoniae carbapenemase; NDM, New Delhi metallo-β-lactamase; NMC, not metalloenzyme carbapenemase; OXA, oxacillinase; PER, Pseudomonas aeruginosa RNL-1; PSE, Pseudomonas specific enzyme; SHV, sulfhydrl reagent variable; SME, Serratia marcescens enzyme; SPM, Sao Paulo metallo- β -lactamase; VEB, Vietnamese extended-spectrum β-lactamase; VIM, Verona integron-encoded metallo-β-lactamase.
